# Extracellular Vesicles: Investigating the Pathophysiology of Diabetes-Associated Hypertension and Diabetic Nephropathy

**DOI:** 10.3390/biology12081138

**Published:** 2023-08-16

**Authors:** Abdel A. Alli

**Affiliations:** 1 Department of Physiology and Aging, College of Medicine, University of Florida, Gainesville, FL 32610, USA; aalli@ufl.edu; Tel.: +1-352-273-7877; 2 Department of Medicine, Division of Nephrology, Hypertension, and Renal Transplantation, University of Florida, Gainesville, FL 32610, USA; 3 Department of Pediatrics, College of Medicine, University of Florida, Gainesville, FL 32610, USA

**Keywords:** hypertension, diabetes, diabetic nephropathy, extracellular vesicles, biomarkers, diagnostic markers, therapeutics, cellular crosstalk, intracellular signaling

## Abstract

**Simple Summary:**

Hypertension may present before or after the onset of diabetes, but in either case, it may increase the risk of developing diabetic nephropathy, the leading cause of end-stage kidney disease. Extracellular vesicles allow for communication between different cell types, the alteration of various signaling pathways, and are involved in disease mechanisms. Various omic studies have revealed that circulating extracellular vesicles and urinary extracellular vesicles are a rich source of biomarkers and markers of the prognosis of specific kidney-associated diseases including diabetes-associated hypertension and diabetic nephropathy.

**Abstract:**

Extracellular vesicles (EVs) include exosomes, microvesicles, and apoptotic bodies. EVs are released by all cell types and are found in biological fluids including plasma and urine. Urinary extracellular vesicles (uEVs) are a mixed population of EVs that comprise small EVs that are filtered and excreted, EVs secreted by tubular epithelial cells, and EVs released from the bladder, urethra, and prostate. The packaged cargo within uEVs includes bioactive molecules such as metabolites, lipids, proteins, mRNAs, and miRNAs. These molecules are involved in intercellular communication, elicit changes in intracellular signaling pathways, and play a role in the pathogenesis of various diseases including diabetes-associated hypertension and diabetic nephropathy. uEVs represent a rich source of biomarkers, prognosis markers, and can be loaded with small-molecule drugs as a vehicle for delivery.

## 1. Introduction

**Figure 1:**
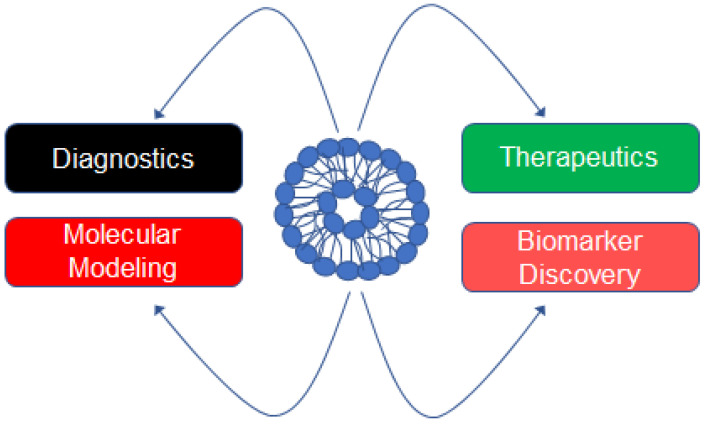
Applications of EVs isolated from biological fluids or cell-conditioned media. EVs can be purified from biological fluids including plasma, serum, urine, or cell-conditioned media. EVs can be used as diagnostic markers and indicate the stage of disease. The molecules enriched in EVs can be subject to bioinformatic studies and used for computational or molecular modeling. Engineered EVs can be loaded with various molecules or drugs for targeted delivery to treat various diseases. EVs are a rich source of biomarkers in physiology and pathophysiology.

Extracellular vesicles (EVs) are a heterogeneous population of vesicles that are generally classified according to their size and biogenesis. Exosomes are the smallest type of EV and originate from the formation of multivesicular bodes [1]. Microvesicles (previously called microparticles) are generally less than 1000 nm in diameter and originate from the budding of the plasma membrane [1]. Apopotic bodies are among the largest type of EV and also originate from the budding of the plasma membrane [1]. EVs were once thought to function as cellular garbage disposals [2], but it is now accepted that EVs play an important role in cellular communication [3,4,5], intracellular signaling [6,7,8], and cellular differentiation [9,10,11]. In addition, EVs are used in multiple applications [12,13,14]. EVs isolated from biological fluids including urine [15], plasma [16], and cerebrospinal fluid [17] represent a rich source of biomarkers. Additionally, the profiling of the EV cargo can be used as markers of diagnosis and prognosis [18,19]. Engineered EVs can be loaded or fused with small-molecule drugs and used as a vehicle for targeted delivery and combination drug therapy [20,21]. One study demonstrated the renoprotective potential of uEVs after showing the loss of miRs through EVs may be associated with a decrease in the levels in the kidney and promote renal fibrosis in diabetic nephropathy [22]. Various applications of EVs are depicted in Figure 1. EVs derived from plasma or urine samples contain important molecules that have been used in the diagnostics of various diseases and in some cases reveal information about different stages of disease [23,24,25,26]. Engineered EVs have been shown to have therapeutic potential [27,28]. In addition, EVs have been studied in the context of computational or molecular modeling [29,30]. The cargo enriched in EVs has also been shown to serve as a rich source of biomarkers for various diseases [31,32,33].

## 2. EVs in Cellular Communication and Alterations of Intracellular Signaling Pathways in Physiology and Pathophysiology

**Figure 2:**
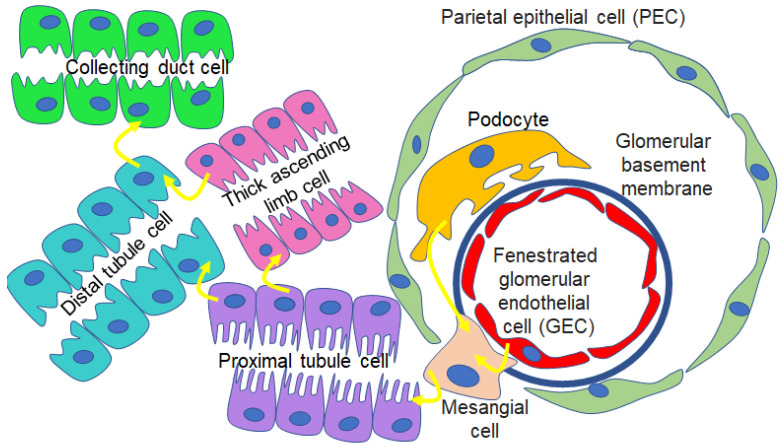
EV-mediated crosstalk between various kidney cell types. EVs mediate intercellular communication between podocytes and mesangial cells, endothelial cells and mesangial cells, glomerular cells and tubular cells, and between other cell types within the segments of the nephron.

EVs have been shown to allow for crosstalk between different cell types, including those within different segments of the nephron (Figure 2). A study by Wu et al. showed that EVs from high-glucose-treated glomerular endothelial cells activate glomerular mesangial cells by promoting proliferation and increasing extracellular-matrix protein production and alpha smooth muscle actin expression [34]. A study by Jella et al. showed that EVs from proximal tubule cells regulate ENaC activity in recipient Xenopus distal tubule cells [3]. Borges et al. showed EVs released from injured tubular epithelial cells shuttle TGF-β1 mRNA and initiate tissue repair and regenerative responses [35]. A study by Jeon et al. showed that EVs released from damaged podocytes are enriched in various microRNAs that promote apoptosis of renal tubular epithelial cells [36]. It is evident that EVs can mediate changes in cellular function in an autocrine- or paracrine-dependent manner. In addition, EVs can modulate gene expression, control biological pathways, and change the phenotype of cells.

The pathophysiology of hypertension and diabetes-associated hypertension is thought to involve crosstalk between different organ systems and cell types, and also changes in intracellular signaling pathways. The crosstalk between various cell types that are affected during the pathophysiology of diabetes and hypertension can be mediated by bioactive and/or signaling molecules enriched in EVs.

## 3. Salt-Sensitive Hypertension and Enrichment of Blood-Pressure-Regulating Proteins in uEVs

The development of salt-sensitive hypertension is thought to be multifactorial with complicated genetic influences. Accordingly, the exact mechanisms that are responsible for the underlying pathogenesis are still not completely understood. However, it is generally accepted that kidney dysfunction plays an important role in the imbalance of fluid and electrolytes, and may contribute to the development of salt-sensitive hypertension. Several renal proteins that play an important role in electrolyte balance and blood pressure regulation including NCC, NKCC2, pendrin, and ENaC were shown to be enriched in human uEVs [37,38,39,40].

Several epithelial transport mechanisms have been studied in the context of the induction and maintenance of salt-sensitive hypertension. Multiple studies have shown db/db mice develop hypertension upon salt-loading [41,42,43,44]. A study by Scindia et al. showed that metformin mitigated high blood pressure in hypertensive diabetic db/db mice in a mechanism involving the attenuation of renal ENaC [43]. A study by Lugo et al. showed that alpha-1 antitrypsin (AAT) treatment by intraperitoneal injection normalized blood pressure in salt-loaded hypertensive diabetic db/db mice in a mechanism involving the attenuation of renal ENaC and MARCKS protein expression [42].

## 4. Association between Circulating EVs and Increased Blood Pressure

Published studies have shown that plasma/serum EVs and urinary EVs are associated with high blood pressure. The data from these studies suggest EVs may be a rich source of biomarkers for the pathogenesis and progression of hypertension. A study by Otani et al. showed that the intraperitoneal injection of plasma EVs from spontaneously hypertensive rats significantly increases systemic blood pressure in Wistar-Kyoto rats with normal blood pressure [45]. Conversely, this group showed that EVs from normotensive Wistar-Kyoto rats prevented an increase in systolic blood pressure in spontaneous hypertensive rats [45]. Additionally, this group also showed that plasma EVs from Wistar-Kyoto rats inhibited the increase in the prostaglandin F2α-induced contraction of the mesenteric artery and prevented perivascular fibrosis in spontaneously hypertensive rats [45]. A study by Good et al. showed that circulating EVs may contribute to the pathogenesis of hypertension and end-organ failure through the disruption of normal vascular tone [46]. This group showed that the functions of both resistance arteries and EVs in the systemic circulation are altered in spontaneously hypertensive rats 12 weeks of age. A study by Sun et al. showed that the circulating levels of endothelial microparticles may reflect renal microvascular capillary injury in patients with hypertension [47]. This group showed that the microparticle levels from renovascular hypertensive patients correlated directly with the histological peritubular capillary count, stenotic kidney hypoxia, and fibrosis, but inversely with cortical perfusion [47].

Previous studies have investigated EV surface biomarkers. Rai et al. investigated the surfaceome of small and large EVs [48]. These surface biomarkers may be important in the context of cellular injury and provide clinical insights into EV function. Moreover, the proteins enriched on the surface of EVs may provide clues to barrier dysfunction. For example, an increase in endothelial microparticles may reflect damage to the endothelium. Also, an increase in small EVs positive for platelet or endothelial cell markers in the urine may indicate a dysfunction of the glomerular filtration barrier.

## 5. Urinary Excretion of Extracellular Vesicles in Diabetes and Diabetic Nephropathy

uEVs are enriched in various lipids, metabolites, and proteins. These vesicles have been used to identify novel biomarkers in the pathophysiology of diabetes. The hydrophilic core and lipid bilayer of EVs protect genetic information from degradation and protects proteins from proteolysis. The packaged cargo of uEVs has been reported to change in conditions of hyperglycemia or high-glucose-induced injury. Multiple studies have identified specific miRNAs, mRNAs, and proteins in uEVs that can be useful in diagnosing diabetic nephropathy. Several studies have profiled various miRNAs in different diabetic patient subgroups or glucose conditions (Table 1). Jia et al. showed that the levels of miR-192, miR-194, and miR-215 are increased in type 2 diabetic patients with microalbuminuria compared to patients with normoalbuminuria and control subjects, but are decreased in patients with macroalbuminuria [49]. Mohan et al. reported that an elevation in renal miR-451-5p and miR-16 may have a protective effect in diabetes-induced renal fibrosis [50]. Prabu et al. demonstrated that miR-30a-5p concentrations were greater in uEVs isolated from macroalbuminuria patients but not patients with T2DM and normoalbuminuria or T2DM and microalbuminuria [51]. Delić et al. showed that miR-320c and miR-6068 are upregulated in uEVs from diabetic nephropathy patients [52]. Barutta et al. showed that miR-145 is increased in mesangial cells and exosomes derived from mesangial cells after high-glucose treatment [53]. Also, the proteins enriched in uEVs in diabetic patients or from diabetic db/db mice treated with various therapeutic agents have been studied. Kalani et al. showed that Wilms’ tumor 1 protein was enriched in uEVs from patients with proteinuria compared to those without proteinuria [54]. In a study by Zubiri et al., regucalcin protein was found to be downregulated in uEVs from rats with diabetic nephropathy [55]. A study by Musante et al. investigated the alterations in protease profiles in uEVs in diabetic nephropathy patients [56]. Lugo et al. showed that multiple hexosylceramides and other classes of lipids are increased in uEVs released from hypertensive diabetic db/db mice compared to control animals [42]. A study by Scindia et al. showed that uEVs from diabetic db/db mice are enriched in the cysteine protease cathepsin B, and the amount is attenuated after treating the mice with metformin compared to the vehicle [43]. In another study by Gholam et al., the concentration of EVs and their size were shown to be less in dapagliflozin-treated diabetic mice compared to vehicle-treated mice [57]. While it has been reported that the concentration of EVs is greater in urine from db/db mice with hyperglycemia or from conditioned media of various cell types [34,58] treated under high-glucose conditions, it appears this is not the case for all cell types. Wen et al. reported that high-glucose conditions reduced EV secretion from mouse kidney proximal tubule BUMPT cells [59]. Interestingly, this study showed that EVs from high-glucose-treated proximal tubule cells activate fibroblasts [59]. This group showed that several proteins were upregulated in the group of cells treated with high glucose compared to normal glucose.

Numerous animal models have been used to study mechanisms associated with the pathogenesis of diabetic nephropathy. The db/db mouse model is perhaps the most characterized and utilized model to study the many aspects that recapitulate human pathophysiology. Also, this mouse model exhibits similar functional and histological features associated with human metabolic dysfunction. These mice present with substantial albuminuria and mesangial matrix expansion. A study by Cohen et al. showed that diabetic nephropathy in db/db mice is ameliorated after inhibiting albumin glycation [60].

## 6. uEVs and Diabetes-Associated Hypertension

There is a high incidence of hypertension in patients with human type 2 diabetes. The presence of hypertension is known to worsen the complications associated with diabetes [61]. Db/db mice have become a popular model to study the various pathways and signaling cascades associated with diabetes-associated hypertension.

The db/db mouse model was established in 1966 by Jackson Labs. In the db/db mouse, there is point mutation in the leptin receptor gene. Mice homozygous for the db mutation are morbidly obese and diabetic within 8 weeks of age. Db/db mice recapitulate many of the symptoms seen in type 2 diabetic patients, including hyperglycemia, insulin resistance, compensatory hyperinsulinemia, and obesity. This animal model has provided important insights into the mechanisms of diabetes and obesity. There are multiple strains of the db/db mouse model, and the course of disease is greatly influenced by the genetic background.

The pathogenesis of disease is most aggressive in the C57BLKS/J background. Multiple studies have investigated blood pressure in db/db mice, and there are some differences between those studies. For example, some studies show that db/db mice have significantly elevated systolic and mean arterial pressures [62,63], while another study showed a reduced arterial pressure in db/db mice compared to wild-type mice [64]. This could be attributed to the different genetic backgrounds of db/db mice, various diets, and the range of ages that were used in those studies. For example, db/db mice used in a study by Su et al. were of a C57BL/KsJ background with a plasma glucose of 537.3 ± 33.3 mg/dL and elevated systolic, diastolic, and mean arterial pressures, while mice used in a study by Bodary et al. [64] had a plasma glucose of 340 mg/dL.

Db/db mice have become an appreciated model to investigate the mechanisms associated with the development of hypertension in the diabetic kidney. These mice develop profound hypertension after salt loading and have elevated ENaC protein expression and proteolysis [42,43]. Also, this model can be useful in investigating the crosstalk between multiple organ systems, including the cardiovascular and renal systems. For example, future studies may investigate how cardiac hormones regulate epithelial transport mechanisms in the kidney.

## 7. Relevance of Protease and Protease Inhibitor Enrichment in EVs to Diabetic Kidney Disease

Various proteases including cathepsins [43,65,66] and prostasin [37] and the versatile human alpha-1 antitrypsin (AAT) protein [67] have been found in EVs. Cathepsin B expression is known to increase with obesity and modulate inflammatory markers [68]. Therefore, it is not surprising that cathepsin B expression was found to be elevated in the kidney of hypertensive diabetic db/db mice that were strikingly obese. Cathepsin S levels are elevated in individuals with type 2 diabetes and obesity, but are reduced after weight loss [69]. Consistent with cathepsin S levels being increased in type 2 diabetes and obesity, the results from a study by Karimkhanloo et al. showed increased cathepsin S plasma levels in db/db mice compared to lean normoglycemic db/+ littermates [70]. In addition, this group showed that cathepsin S regulated glucose metabolism in cultured primary hepatocytes that were isolated from db/db mice but found that acute cathepsin S administration did not affect the plasma insulin levels or glucose tolerance of diabetic db/db mice [70].

A study by Hadler-Olsen showed there was an increased amount of gelatin-degrading serine proteinases in the plasma of db/db mice compared to db/+ control mice [71]. This study also showed that there was a greater amount of MMP-9 (gelatinase B) in the kidney extracts of db/+ control mice compared to db/db mice. Conversely, the expression of MMP-9 in the kidneys of KKay mice that developed nephropathy was augmented when compared to its expression in control mice.

The efficacy of exogenous kallikrein in ameliorating the effects of diabetic nephropathy in db/db mice was reported by Liu et al. [72]. In that study, inflammation, renal fibrosis, and oxidative stress were ameliorated in diabetic mice treated with kallikrein compared to diabetic controls [72]. In addition, exogenous kallikrein resulted in a decreased glomerular basement membrane thickness and protected against the loss of endothelial fenestrae, loss of podocytes, the effacement of foot processes in diabetic mice, and ultimately mitigated diabetic nephropathy [72].

## 8. Contribution of EVs in the Development of Insulin Resistance and Diabetes and Associated Complications

A study by Kumar et al. showed exosomes isolated from obese mice fed a high-fat diet or from type 2 diabetic patients caused lean mice to develop insulin resistance [73]. This group showed that CD63+A33+ exosomes are released from the intestinal epithelial cells of high-fat mice, and the uptake of these exosomes by liver cells is mediated by exosomal lipid composition. Moreover, they showed that the uptake of exosomes from high-fat-diet-fed mice by liver macrophages leads to the release of IL-6 and TNF-α. The results from this study showed that the CD63+A33+ exosomes induce insulin resistance and glucose intolerance in a mechanism involving the ligand-activated transcription factor AhR. A study by Abdelsaid et al. showed that plasma exosomes isolated from diabetic mice reduced angiogenic responses, while plasma exosomes from exercise trained T2DM mice restored the angiogenic effects in endothelial cells and in wound-healing models [74]. This group showed the mechanism by which exosomes mediated their effects involved an increase in the copper transporter ATP7A and the superoxide dismutase SOD3 protein in the plasma exosomes [74]. A study by Pillai et al. showed that the oxidation of LDL results in an increase in exosome secretion by adipocytes allowing for crosstalk with macrophages and the contribution of atherogenesis [75].

## 9. Circadian Regulation of EV Cargo Relevant to Diabetes Research

The mammalian circadian clock regulates many physiological and pathophysiological processes during light and dark cycles. Circadian rhythmicity is maintained by the positive regulators, circadian locomotor output cycles kaput (CLOCK) and brain and muscle aryl hydrocarbon receptor nuclear translocator-like 1 (BMAL1), and their co-repressors, period (PER) and cryptochrome (CRY). Importantly, the cargo enriched in EVs released by various cell types appears to vary over a 24 h cycle. Multiple studies have shown EV marker proteins are differentially enriched in uEVs isolated from urine samples during the inactive and active cycles of adult mice. A study by Lugo et al. showed differences in the amount of annexin A2, GAPDH, TSG101, and caveolin-1 proteins, as well as lipids including specific hexosylceramides, monoacylglycerols, phosphatidylcholines, lysophosphatidylethanolamine, phosphatidylethanolamine, and phosphatidylglycerol enriched in uEVs from male and female diabetic db/db mice treated with human AAT (hAAT) or the vehicle [42]. A study by Lopez et al. showed differences in the amounts of syntein and flotillin proteins, as well as lipids including specific phosphatidylethanolamines, triacylglycerols, and phosphatidylcholines enriched in uEVs from aged male mice with spontaneous hypertension [15]. Yeung et al. performed a functional enrichment analysis of circadian proteins and showed actin-binding and intermediate filament proteins associated with the cytoskeleton had peak abundances in small EVs that were temporally separated at 8 h and ~23 h after synchronization [76]. This group also showed that the various proteins that contribute to vesicle formation had peak abundances between 12 and 24 h after synchronization. Taken together, these studies suggest the biogenesis and packaging of cargo in uEVs are regulated in a circadian-dependent manner. Multiple groups have investigated the cargo enriched in EVs in a time-of-day-dependent manner. A study by Koritzinsky et al. suggested that the circadian variation in EV release can be normalized to TSG101 enrichment or by vesicle number [77]. Su et al. showed that hypertensive diabetic db/db mice have a disrupted heart rate, blood pressure, and locomotor activity that is associated with dampened oscillations of clock genes [78]. Grosbellet et al. showed a lengthened endogenous period altered photic integration in db/db mice [79]. Time-of-day variations in the vasocontractility of db/db mice have been previously investigated. Su et al. showed that normal time-of-day variations in vasocontractility to angiotensin II, phenylephrine, and high-potassium depolarization are lost in the aorta of db/db mice, and this is associated with alterations in the expression of clock genes or their oscillation phases [63]. Multiple groups have shown db/db mice display a nondipping phenotype [78,80,81]. It was suggested that this nondipping blood pressure phenotype in db/db mice is associated with the variations in vasocontractility and the disruption in vascular clock oscillation in these mice [63].

## 10. Renal Handling of EVs

Although there have been significant advances in the treatment of diabetic nephropathy in the past decade, it remains the leading cause of end-stage kidney disease. Alterations in cell types within the glomerulus is important in the pathogenesis of diabetic nephropathy [82].

**Figure 3:**
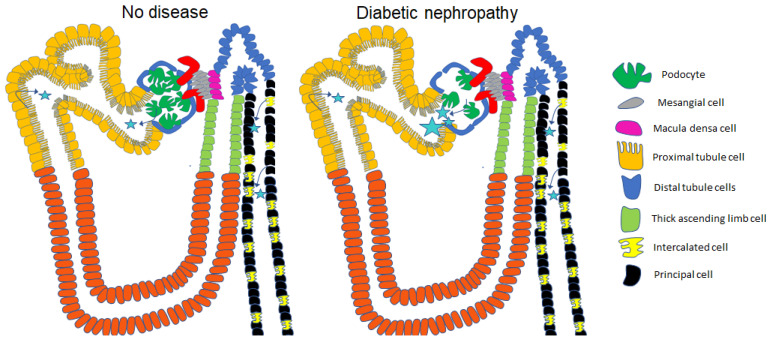
A greater number of EVs from the systemic circulation is filtered across the glomerular filtration barrier with the progression of diabetic nephropathy compared to the health kidney in the absence of disease. Stars represent EVs. Arrows represent the cell types that release EVs into the lumen.

The glomerulus consists of a glomerular basement membrane, fenestrated endothelium, and podocytes with distinct foot processes that allow for highly selective filtration. Various studies suggest that the cutoff for passive glomerular filtration is about 4.4–7.6 nm [83] or 30–50 kDa [84]. Due to the composition of these layers, large molecules and negatively charged molecules are not readily filterable. Molecular composition, size, and charge are important determinants of glomerular filtration. Interestingly, it has been shown that the renal elimination of specific molecules that are relatively large and have negative charges can be filtered and excreted [84]. Although most EVs that are present in the systemic circulation, including larger exosomes and microvesicles, are generally not filterable, smaller EVs may be filtered. The amount of EVs from the systemic circulation that is filtered likely increases when the glomerular filtration barrier is compromised as in diabetic nephropathy, as depicted in Figure 3. Also, larger EVs are filtered, and are either taken up by various cell types within the nephron or excreted in diabetic nephropathy, where there is a dysfunctional glomerular filtration barrier due to podocyte injury, foot process effacement, and/or podocyte loss, which contributes to the increased filtration of circulating EVs, as illustrated in Figure 3. EVs that are present in the tubule lumen can be taken up by any cell type in the nephron leading to cellular crosstalk and changes in intracellular signaling.

Polarized epithelial cells of the kidney that form the different segments of the nephron have an apical plasma membrane that faces the tubular lumen and a basolateral plasma membrane that faces the peritubular capillaries. All cell types in the nephron release EVs across the luminal/apical plasma membrane. However, it is unclear whether EVs are taken up by the peritubular capillaries after being released across the basolateral plasma membrane. In vitro studies imply EVs can be released from the luminal/apical plasma membrane or basolateral plasma membrane. For example, mouse cortical collecting duct cells grown on permeable supports were shown to release EVs across the luminal plasma membrane and the basolateral plasma membrane, and these two populations of EVs were found to be remarkably different in their lipid and protein composition [85]. These two populations of EVs that were released from the same mouse cortical collecting duct cells showed differential enrichment of proteins and lipids. Presumably, other cell types in the nephron release EVs with unique proteomic and lipidomic profiles. The sorting of urinary EVs and plasma EVs, and the characterization of the molecular cargo enriched in these EVs, from an animal model used to study pathophysiology, such as diabetic db/db mice, may allow for the identification of novel biomarkers important in disease mechanisms.

## 11. Proteomics of uEVs and EVs Released from Renal Cell Types

Specific proteins have been shown to be enriched in EVs isolated from glomerular cells and tubule cells (Table 2). Also, the phosphoproteomic profiling of uEVs has been reported [86]. Several proteins associated with renal diseases and hypertension have been identified and discussed in a review by Pisitkun et al. [40]. These proteins include aquaporin-2, epithelial sodium channel subunits, sodium chloride co-transporter, sodium potassium chloride co-transporter 2, carbonic anhydrase II and IV, polycystin-1, uromodulin, podocin, adenine phosphoribosyltransferase, nonmuscle myosin heavy chain IIA, FXYD domain-containing ion transport regulator-2, dimethylarginine dimethylaminohydrolase-1, neprilysin, hydroxyprostaglandin dehydrogenase, angiotensin I-converting enzyme isoform-1, and aminopeptidase N, P, and A. As summarized in Table 2, several studies have identified proteins enriched in EVs released from specific cell types including podocytes, proximal tubule cells, and principal cells [59,85,87,88,89].

## 12. uEVs as Biomarkers for Various Human Diseases

Interestingly, the quantification of specific apical membrane proteins in uEVs is not limited to renal diseases and hypertension. A study by Hayakawa et al. suggested that the quantification of ENaC gamma normalized to CD9 may be a biomarker for mineralocorticoid receptor (MR) activity in primary aldosteronism patients and perhaps other MR-associated diseases [90]. A study by Hu et al. suggested proteolytically cleaved products of ENaC alpha and gamma subunits normalized to CD9 were significantly enriched in uEVs from women with pre-eclampsia compared to those with a normal pregnancy [91], Additionally, the same study showed a phosphorylated form of NCC, at a T60 SPAK/OSR1 site, was significantly reduced in uEVs normalized to CD9 in the pre-eclampsia group compared to the normal pregnancy group [91]. A study by Wu et al. showed that mannan-binding lectin-associated serine protease 2 (MASP2) enrichment in uEVs from immunoglobulin A (IgA) nephropathy (IgAN) patients correlated with the level of urinary microalbumin and suggested MASP2 enrichment in uEVs may be a valuable biomarker for assessing the severity of renal injury as well as the prognosis of IgAN [92]. Taken together, these studies suggest uEVs may serve as noninvasive sources of novel biomarkers that can complement existing methods for diagnosing various diseases in humans. 

## 13. Conclusions

The profiling of EVs isolated from the conditioned media of specific renal cell types cultured under high-glucose conditions or from the urine of animals or patients with hypertension coupled to diabetes or diabetic nephropathy may offer clues to the pathophysiology of these diseases. EVs released from various cell types in response to different stimuli or during the progression of diabetes-associated hypertension or diabetic nephropathy are packaged with bioactive cargo including metabolites, lipids, proteins, mRNAs, and miRNAs that contribute to cellular communication and changes in intracellular signaling. The isolation of plasma EVs and urinary EVs represent a rich source of biomarkers and prognosis markers, while engineered EVs may allow for the delivery of small drugs. The growing number of studies in EV research related to diabetes suggest these vesicles have great potential as tools to help elucidate the complex mechanisms associated with this disease.

## Figures and Tables

**Table 1:** miRs in diabetes and diabetic nephropathy.

mIR	Finding	Reference
miR-192, miR-194, miR-215	increased in type 2 diabetic patients with microalbuminuria compared to patients with normoalbuminuria	[49]
miR-451-5p, miR-16	protective effect in diabetes-induced renal fibrosis	[50]
miR-30a-5p	greater in uEVs isolated from macroalbuminuria patients but not patients with T2DM and normoalbuminuria or T2DM and microalbuminuria	[51]
miR-320c, miR-6068	upregulated in uEVs from diabetic nephropathy patients	[52]
miR-145	increased in mesangial cells and exosomes derived from mesangial cells after high-glucose treatment	[53]

**Table 2:** Enrichment of various proteins in EVs released from specific kidney cell types.

Cell Type	Podocytes	Mesangial Cells	Glomerular Endothelial Cells	Proximal Tubule Cells	Principal Cells
	Podocalyxin, Nephrin (Prunotto et al. [89]) Proteasome subunit beta type-2, Cullin-2, Complement C3, Cystatin-A, Keratin, type I cytoskeletal 14, Putative phospholipase B-like 2, Endoplasmic reticulum resident protein 44 (Barreiro et al. [87])	Creatine kinase, Cysteine-rich motor neuron 1 protein (Barreiro et al. [87])	EGF containing fibulin-like extracellular matrix protein 1 isoform 1, Prosaposin, Pentraxin-related protein, Latenttransforming growth factor beta-binding protein 2, Thioredoxin reductase 1, cytoplasmic, Chondroitin sulphate proteoglycan 4 (Barreiro et al. [87])	CD63, CD9, CD81, TSG101, ALIX, VPS4B, HSP70, HSP84, 60S ribosomal protein L27, V-set domain-containing T-cell activation inhibitor 1, Protein lin-7 homolog C, HLA class I histocompatibility antigen, A-24 alpha chain, TNFAIP3-interacting protein 1, Laminin subunit alpha-3, Beta (β)-2-macroglobulin, Interferon-induced transmembrane protein 1 TNF alpha-induced protein 3, Myosin-10, Serum amyloid A-1 protein, Intercellular adhesion molecule 1, C-X-C motif chemokine 10, Alcohol dehydrogenase, Hyaluronan and proteoglycan link protein 3, Guanylate-binding protein 5, Indoleamine 2,3-dioxygenase 1, Tryptophan–tRNA ligase, cytoplasmic 1, Interferon-induced guanylate-binding protein 1, Tumour necrosis factor receptor superfamily member 5, ADP-ribosyl cyclase/cyclic ADP-ribose hydrolase 1, Tumour necrosis factor alpha-induced protein (Wang et al. [88]) Annexin A5, Annexin A11, Adenylyl cyclase 7, GTPase-activating protein subunit alpha-2, Cofilin-2, Heat shock cognate 71 kDa protein, Phosphoglycerate kinase 1, 14-3-3 protein theta, 14-3-3 protein gamma, 14-3-3 protein zeta/delta, retinoic acid-induced protein 3 (Wen et al. [59]) Betahexosaminidase subunit beta, Elongation factor 1-gamma, Serine/threonineprotein phosphatase PP1-alpha catalytic subunit, Serine/threonineprotein phosphatase PP1-beta catalytic subunit, Brain acid soluble protein 1 (Barreiro et al. [87])	α-actinin-1, moesin, 14-3-3 protein ζ/δ, annexin A1/A3/A4/A5/A6, clathrin heavy chain 1, GAPDH, α-enolase, filamin-A, HSP 90 (Dang et al. [85])

## Data Availability

No unpublished data are referenced in this review.
